# Associations between COVID-19 incidence, weight status, and social participation restrictions in the U.S.: evidence from the national population, cross-sectional study

**DOI:** 10.1186/s12889-024-18566-y

**Published:** 2024-04-17

**Authors:** SuJung Jung

**Affiliations:** School of Nursing, California State University Long Beach, Long Beach, USA

**Keywords:** COVID-19, Weight status, BMI, Social participation restrictions

## Abstract

**Background:**

To explore the associations between coronavirus infection incidence and weight status and social participation restrictions among community-dwelling adults in the United States.

**Methods:**

We analyzed data from the 2021 National Health Interview Survey (NHIS), which included a representative sample of 29,394 individuals (Coronavirus disease 2019 (COVID-19): 3,205) and a weighted total of 252,461,316 individuals (COVID-19: 31,697,404), considering the complex sampling design used in the survey.

**Results:**

Age, race/ethnicity, education level, family income index, body mass index (BMI), and smoking status were significantly associated with COVID-19 infection. Weight status was significantly correlated with social participation restrictions and strongly associated with COVID-19 infection, particularly among individuals who were overweight or obese.

**Conclusion:**

Weight status was shown to be associated not only with social participation restrictions but also with COVID-19 infection among U.S. adults. Understanding the complex interplay between weight status, social participation, and COVID-19 is crucial for developing effective preventive measures and promoting overall well-being in the community population.

## Background

The novel coronavirus has rapidly spread to millions of individuals worldwide, resulting in significant morbidity and mortality [1]. The COVID-19 pandemic has brought about a profound transformation in global health. To address the escalating transmission rates, authorities at the federal, state, and local levels in the U.S. have implemented targeted public health interventions with the aim of controlling the spread of the virus effectively [2]. The Centers for Disease Control and Prevention estimates that the impact of COVID-19 on the United States corresponds to approximately 1 in every 1.32 COVID-19-related deaths, highlighting the significant burden imposed by the disease [2].

The potential relationship between weight and COVID-19 incidence has been acknowledged by the scientific community and healthcare professionals. There is a strong association between obesity and a significant increase in the incidence of illness and death related to COVID-19 in individuals [3]. Obesity is also a major public health concern in the United States, imposing an annual cost of approximately $173 billion on the healthcare system [4]. In the United States, approximately 30.2% of adult hospitalizations due to COVID-19 were directly attributed to obesity (body mass index ≥30 kg/m^2^) [5]. Additionally, countries with higher BMI levels exhibit elevated rates of both COVID-19 incidence and mortality [6]. Specifically, a higher body mass index is linked to an increased risk of COVID-19-related hospitalization and death in the United States [7].

Social participation refers to engaging in activities that involve interacting with others in a society or community [8], and it has been linked to various health outcomes, including mortality, morbidity, and quality of life [8]. However, the implementation of social distancing measures during the pandemic, necessary for individual health and safety, poses a challenge to social participation. Consequently, individuals may choose to stay at home and refrain from going out, leading to a decrease in participation in social activities This trend raises new concerns about the complex relationship between health and social interaction. Specifically, previous findings indicated an interplay between social participation and obesity. For example, a systematic literature review provided evidence of the association between social participation and obesity [9]. Additionally, there was a noticeable increase in sedentary behavior and excessive eating during the lockdown, leading to an imbalance in energy intake and subsequent weight gain [10]. These findings underscore the critical need to comprehend the dynamic between weight status and social participation during the pandemic.

Globally, having a high BMI (metabolic risk) was associated with death from diabetes and disability-adjusted life years [11]. However, previous studies have faced limitations in identifying unmeasured variables that could affect metabolic control due to data constraints [12, 13]. Furthermore, previous studies have relied primarily on medical records, which has led to a limited understanding of weight status, social participation restrictions, and COVID-19 infection among individuals in the community.

The objective of this study was to investigate the incidence of COVID-19 and its association with weight status and social participation restrictions in adults within the U.S. community utilizing data from the 2021 National Health Interview Survey. Specifically, our aims were to analyze the prevalence of COVID-19 incidence, explore the relationship between COVID-19 incidence, weight status, and social participation restrictions, and identify potential risk factors associated with its occurrence. By investigating these factors within a population-based framework, our study contributes to a better understanding of the broader transmission of COVID-19 and its implications for future public health interventions.

## Methods

### Study design and subjects

For our study, we utilized data from the National Health Interview Survey, a long-standing survey implemented since 1957 to monitor the health of the nation. The 2021 NHIS data, covering COVID-19 status measurements from January to December 2021, formed the foundation of our analysis [14]. To prioritize safety during face-to-face interview, the survey implemented several protocols such as wearing masks, practicing social distancing, conducting interviews outdoors, refraining from entering respondent’s homes, and prohibiting respondents from touching Census Bureau equipment or materials [15]. Out of the 29,394 participants aged ≥18 who were asked about their positive COVID-19 diagnosis based on the test, 3,205 tested positive for COVID-19. Our study conducted a secondary data analysis using publicly available de-identified NHIS data. As a result, no Institutional Review Board approval was necessary [16, 17].

### Outcome variable: COVID-19 incidence

Participants who answered “yes” to the questions “Have you ever been tested for coronavirus or COVID-19?”, “Did the test confirm that you had coronavirus or COVID-19?”, and “yes” to this question were classified as confirmed COVID-19 cases [14].

### Exposure variables: social participation restrictions, weight status, smoking status, diabetes status, hypertension status, and history of heart attack

We investigated social participation restrictions, weight status, smoking status, diabetes status, hypertension status, and history of heart attack. Individuals who indicated having “a lot of difficulty” or being “can’t do at all” in response to the following question were categorized as having social participation restrictions: “Because of a physical, mental, or emotional condition, do you have difficulty participating in social activities such as visiting friends, attending clubs and meetings, or going to parties?” [18]. Weight status was measured using BMI. The NRS-2002 is used to evaluate overweight and obesity and is calculated by dividing the participant’s weight in kilograms by the square of their height in meters. Individuals with a BMI of 25 kg/m^2^ or higher were categorized as overweight, while those with a BMI of 30 kg/m^2^ or higher were classified as obese. Participants were asked a series of questions about their smoking habits and status. The questions included the following: (1) “Do you smoke now?”, (2) “Do you smoke every day, some days, or not at all?”, and (3) “Have you ever smoked 100 cigarettes in your lifetime?” Based on their responses, participants were categorized as follows: those who reported currently smoking, smoking daily, or smoking occasionally during the survey were classified as current smokers; individuals who had smoked 100 cigarettes in the past but were not currently smoking were classified as former smokers; and participants who had never smoked were classified as never smokers [19]. The presence of diabetes, hypertension, or heart attack was determined based on the participants’ response to the question "Have you ever been told by a doctor or health professional that you had hypertension, diabetes or heart attack?" Participants who answered affirmatively to any of these conditions were classified accordingly.

### Covariates

To address potential confounding factors, we controlled for several covariates, including age (categorized as 18-59 and 60 years or older), sex (male and female), race/ethnicity (classified as white only, black/African American only, American Indian/Alaska native only, Asian only and the other race/ethnicity group), educational attainment (divided into four categories: (a) less than high school education (<12th grade or a general educational development program), (b) high school graduate, (c) associate degree (some college, associate degree, occupational, technical, or vocational program), and (d) bachelor’s degree or higher (bachelor’s or master’s degree, or doctoral degree)), and family income index [20].

### Statistical analysis

We estimated the actual number of reported COVID-19 cases as well as the weighted number of cases. To examine differences in participant characteristics, we conducted Pearson’s chi-square test. Spearman’s correlation analysis was then employed to evaluate the correlations between COVID-19 infection incidence and weight and social participation restrictions. Finally, we used logistic regression models to estimate adjusted odds ratios (ORs) of predictors of COVID-19. All the statistical analyses were conducted using SPSS version 28.0, and a significance level of 0.05 (two-tailed) was applied.

## Results

Table 1 shows that 3205 patients were classified as COVID-19, corresponding to an estimated total of 31697404 COVID-19 patients. There were significant differences in the characteristics of the COVID-19 patients based on age, race/ethnicity, level of education, family income index, BMI, and smoking status. Individuals aged 18-59 exhibited a higher percentage of COVID-19 cases compared to those aged 60 or above, with a significant association found between age group and COVID-19 infection. Similarly, significant associations were observed between race/ethnicity, marital status, education level, and family income index with COVID-19 infection, excluding sex. While social participation restrictions did not show significant association with COVID-19, BMI and smoking status did. In particular, overweight (BMI 25<=BMI<30/m^2^) and obese (BMI>=30 kg/m^2^) individuals had significantly greater COVID-19 incidence than did their corresponding counterparts.

**Table 1:** COVID-19 cases according to participant characteristics

		COVID-19 (*n*=3205/31697404)			Chi-square test	*p* value
		Positive case	%^a^ (weighted)	Weighted case		
Age, years	18-59	2336	20.7 (22.5)	25084149	105.872	<.001
	60 or above	869	14.4 (16.1)	6613255		
	Mean (SD)/weighted	52.50 (18.353)/48.12(18.454)				
Sex	Male	1409	18.5 (20.4)	12498688	.016	.901
	Female	1796	18.5 (21.1)	17098717		
Race/ethnicity	White only	2360	18.6 (20.9)	22766094	30.209	<.001
	Black/African American only	378	17.8 (19.5)	3929055		
	American Indian/Alaska native only	28	19.0 (26.9)	371459		
	Asian only	117	11.7 (12.4)	1035132		
	other	75	18.4(18.3)	651917		
Marital status	Married	1553	19.4 (21.2)	16283589	21.495	<.001
	Never married	862	19.0 (21.1)	9822080		
	Divorced/widowed/separated	684	16.1 (17.9)	4467898		
Education	Less than high school	292	22.2 (25.1)	3191888	149.116	<.001
	High school	898	23.0 (25.7)	10309733		
	Associate degree	949	19.9 (21.9)	8899922		
	College or above	1048	14.5 (15.6)	9108406		
Family income index	0-0.99	338	20.9 (24.8)	3584969	48.721	<.001
	1-1.74	452	21.9 (25.1)	4587220		
	1.75-2.99	692	20.4 (22.6)	7009102		
	3 or above	1723	16.8 (18.6)	16516114		
SPR	Yes	108	17.7 (19.8)	998728	.371	.542
	No	3094	18.5 (20.8)	30682518		
BMI	Under weight (<18.5)	39	14.9 (18.4)	468188	54.277	<.001
	Normal weight (18.5<=BMI<25)	831	15.7 (17.9)	3277390		
	Overweight (25<=BMI<30)	1094	18.8 (21.0)	10616928		
	Obesity (BMI=>30)	1176	21.1 (23.5)	11729311		
Smoking	Yes	1052	17.1 (19.1)	9303673	12.615	<.001
	No	2063	19.3 (21.5)	21442323		
DM	Yes	2855	18.4 (21.6)	3100364	.978	.323
	No	348	19.4 (20.7)	28583758		
Hypertension	Yes	1100	18.1 (20.5)	9557507	1.252	.263
	No	2102	18.8 (20.9)	22112276		
Heart attack	Yes	107	17.1 (20.5)	939011	.839	.360
	No	3097	18.6 (20.8)	30755959		

*COVID-19* Coronavirus disease 2019, *SD* Standard deviation, *SPR* Social participation restrictions, *BMI* Body mass index, *DM* Diabetes mellitus

Missing data were excluded

^a^Among those who reported that they had COVID-19 testing

Table 2 displays the correlations between COVID-19 infection incidence and weight status and social participation restrictions. There was a strong correlation between COVID-19 incidence and weight status (*r*=0.056, *p*=<.001). In addition, weight status was significantly correlated with social participation restrictions (*r*=0.012, *p*=.043). However, there was no significant correlation between COVID-19 infections and social participation restrictions.

**Table 2:** Correlations between COVID-19 incidence, weight status and social participation restrictions

	COVID-19 infections	Weight status	Social participation restrictions
	*r* (*p*)	*r* (*p*)	*r* (*p*)
COVID-19 infections			
Weight status	.056 (<.001)		
Social participation restrictions	-.005 (.542)	.012 (.043)	

*COVID-19* Coronavirus disease 2019

Table 3 shows that the odds analysis revealed significant associations between exposure factors and COVID-19 infection risk. In terms of BMI, individuals classified as overweight exhibited a significantly higher likelihood of COVID-19 infection, with obese individuals showing and even greater risk. In addition, smoking was significantly associated with COVID-19 infection risk. However, health conditions such as DM, hypertension, and heart attack did not exhibit statistically significant associations with COVID-19 infection risk.

**Table 3:** Factors associated with COVID-19 infection

		OR	(95% CI)	*p* value
SPR (ref. no)	Yes	.907	(.717-1.148)	.418
BMI (ref. normal)	Underweight	.894	(.616-1.296)	.554
	Overweight	1.220	(1.095-1.359)	<.001
	Obesity	1.309	(1.173-1.461)	<.001
Smoking (ref. never smoked)	Ever smoked	.832	(.759-.911)	<.001
Health conditions	DM	1.065	(.921-1.232)	.359
	Hypertension	1.083	(.978-1.200)	.126
	Heart attack	.958	(.756-1.214)	.722

COVID-19 Coronavirus disease 2019, *OR* Odds ratio, *SPR* Social participation restrictions, *BMI* Body mass index, *DM* Diabetes mellitus

Covariates (age, sex, race/ethnicity, marital status, education, and family income index) were adjusted

## Discussion

This study aimed to explore the associations between COVID-19 incidence, weight status, and social participation restrictions in the U.S. The main results showed that weight status was associated with social participation restrictions and COVID-19 infection. Specifically, individuals who were overweight or obese were found to be more susceptible to coronavirus infection, as evidenced by national data in the U.S. As the first study to examine the association of these variables using a nationally representative dataset, the findings of this study have important implications for public health interventions and strategies.

The study revealed a significant association between weight status and social participation restrictions in U.S. adults. Individuals with a higher BMI were more likely to experience limitations in their engagement in social activities. This association can be attributed to the social stigma faced by individuals with larger body sizes in the U.S. [21], which can lead to social isolation [22]. Moreover, overweight or obesity can hinder individuals’ participation in various social activities, such as joining local sports teams or engaging in physical activities in public spaces [23]. The COVID-19 pandemic has further exacerbated these social restrictions through lockdown measures [24]. In addition, obesity plays a role in creating differences in overall and healthy life expectancy for the U.S. population [25]. To promote a healthy post-COVID life, it is necessary to implement measures that facilitate social engagement for individuals who are overweight or obese.

In addition, the study emphasized the association between weight and COVID-19 infection, which was supported by public data. Individuals with a higher body weight index were shown to be at an elevated risk of contracting COVID-19. These findings are consistent with emerging evidence suggesting that obesity is a significant risk factor for severe outcomes related to COVID-19 [5, 7]. During the pandemic, obesity has been shown to be an independent risk factor for increased hospitalization and mortality compared to a normal BMI. Researchers have explained that the pathophysiological understanding of this association involves the inflammatory state associated with obesity [26]. Another concern is that individuals with obesity often have comorbidities [27], which further increase their vulnerability to COVID-19 [28]. Additionally, high BMI and obesity have been identified as risk factors for the development of long-term COVID-19 [29, 30], which refers to persistent symptoms following the acute phase of the infection. Given these findings, addressing obesity as a public health priority and implementing targeted preventive measures for high-risk populations are crucial.

One notable finding of our study is the lack of significant association between social participation restriction and COVID-19 infection in our regression and correlation models. While this may seem counterintuitive, it underscores the complexity of factors influencing COVID-19 transmission [31]. Social participation restrictions, although implemented as preventive measures, may not directly correlate with infection rates due to the presence of cofounding variables or other mediating factors that were not fully accounted for in our analysis. Future research should explore additional factors that may mediate or moderate these relationships using additional data sources, which may provide further insights into the relationship between social participation restriction and COVID-19 infection. In addition, while the finding that smokers were less likely to be infected by COVID-19 than never-smokers is noteworthy, it should be interpreted within the broader context of the detrimental effects of smoking on overall health [32]. Moreover, there are studies that have highlighted the ongoing debate regarding the relationship between smoking and vulnerability to COVID-19 [33]. Therefore, further research is needed to understand the mechanisms underlying this observed association and its implications for public health policy and practice.

### Strengths and limitations

This study contributes to our understanding of weight status, social participation restrictions and COVID-19 infection in several significant ways. First, this study provides a national estimate of the burden of COVID-19 among adults residing in the U.S. in 2021. Second, the study revealed a noteworthy association between weight status and the risk of COVID-19, indicating that effectively managing weight status may reduce the risk of COVID-19. These findings emphasize the importance of developing interventions and policies at the community level, targeting high-risk populations, to address weight and its impact on COVID-19 incidence. Third, the study sheds light on the relationship between weight status and social participation restrictions, emphasizing the need to raise awareness about weight bias and promote education and empathy to reduce social stigmatization toward individuals with larger body sizes. Additionally, this study underscores the significance of recognizing the potential psychological impact of social participation restrictions and providing resources for mental health support.

This study has certain limitations that should be acknowledged. First, the use of self-reported data for the variables introduces the possibility of recall bias and measurement error. Second, the cross-sectional design of the study prevents the establishment of causality or determination of the temporal sequence of events. Conducting longitudinal studies could provide more robust evidence regarding the observed associations. Finally, although the study claims to use nationally representative data, it is important to consider the generalizability of the findings beyond the U.S. population. The relationships between weight status, social participation restrictions, and COVID-19 infection may vary according to culture, socioeconomic status, and healthcare context in other countries. Despite these limitations, the findings of this study, based on nationally representative data, contribute valuable new evidence regarding the association between weight status, social participation restrictions, and COVID-19 incidence. The use of a representative population survey helps to avoid the risk of collider bias [34]. Unlike previous studies that focused on individuals admitted to hospitals with COVID-19 symptoms, which may introduce sampling bias, this study benefits from the use of national survey data, providing more reliable evidence.

## Conclusion

This study offers important insights into the association between COVID-19 incidence, weight status, and social participation restrictions in the U.S. The findings demonstrated that weight status is linked to both social participation restrictions and COVID-19 infection, with overweight and obesity being particularly associated with increased susceptibility to the virus. This finding emphasizes the need to address the impact of weight status on social engagement and COVID-19 infection and advocates for public health interventions targeting weight management and the reduction of social stigmatization. These findings may have significant implications for public health strategies.

## Data Availability

All data generated or analyzed during this study is publicly-available dataset. Data was available in https://www.cdc.gov/nchs/nhis/2021nhis.htm website.

## Abbreviations

COVID-19: Coronavirus disease 2019
BMI: Body Mass Index
NHIS: National Health Interview Survey
DM: Diabetes mellitus
